# Multimodal Breast Phantoms for Microwave, Ultrasound, Mammography, Magnetic Resonance and Computed Tomography Imaging

**DOI:** 10.3390/s20082400

**Published:** 2020-04-23

**Authors:** Giuseppe Ruvio, Raffaele Solimene, Antonio Cuccaro, Gaia Fiaschetti, Andrew J. Fagan, Sean Cournane, Jennie Cooke, Max J. Ammann, Jorge Tobon, Jacinta E. Browne

**Affiliations:** 1 School of Medicine, National University of Ireland Galway, Galway 8, Ireland; 2 Endowave Ltd., Dublin 2, Ireland; 3 Dipartimento di Ingegneria, Università degli Studi della Campania Luigi Vanvitelli, 81031 Aversa, Italy; raffaele.solimene@unicampania.it (R.S.); antonio.cuccaro@unicampania.it (A.C.); 4 Department of Information Engineering, Electronics, and Telecommunications, Sapienza University, 00185 Rome, Italy; gaiafiaschetti@gmail.com; 5 Department of Radiology, Mayo Clinic, Rochester, MN 55905, USA; Fagan.andrew@mayo.edu (A.J.F.); browne.jacinta@mayo.edu (J.E.B.); 6 Medical Physics and Bioengineering Department, St. James’s Hospital, Dublin 8, Ireland; S.Cournane@st-vincents.ie (S.C.); JCooke@stjames.ie (J.C.); 7 Antenna & High Frequency Research Centre, Technological University Dublin, Dublin 8, Ireland; max.ammann@tudublin.ie; 8 DET—Department of Electronics and Telecommunications, Politecnico di Torino, 10129 Torino, Italy; jorge.tobonvasquez@polito.it; 9 School of Physics and Clinical & Optometric Sciences, Medical Ultrasound Physics and Technology Group, IEO, FOCAS, Technical University Dublin, Dublin 8, Ireland

**Keywords:** microwave imaging, dielectric properties, tissue-mimicking materials

## Abstract

The aim of this work was to develop multimodal anthropomorphic breast phantoms suitable for evaluating the imaging performance of a recently-introduced Microwave Imaging (MWI) technique in comparison to the established diagnostic imaging modalities of Magnetic Resonance Imaging (MRI), Ultrasound (US), mammography and Computed Tomography (CT). MWI is an emerging technique with significant potential to supplement established imaging techniques to improve diagnostic confidence for breast cancer detection. To date, numerical simulations have been used to assess the different MWI scanning and image reconstruction algorithms in current use, while only a few clinical trials have been conducted. To bridge the gap between the numerical simulation environment and a more realistic diagnostic scenario, anthropomorphic phantoms which mimic breast tissues in terms of their heterogeneity, anatomy, morphology, and mechanical and dielectric characteristics, may be used. Key in this regard is achieving realism in the imaging appearance of the different healthy and pathologic tissue types for each of the modalities, taking into consideration the differing imaging and contrast mechanisms for each modality. Suitable phantoms can thus be used by radiologists to correlate image findings between the emerging MWI technique and the more familiar images generated by the conventional modalities. Two phantoms were developed in this study, representing difficult-to-image and easy-to-image patients: the former contained a complex boundary between the mammary fat and fibroglandular tissues, extracted from real patient MRI datasets, while the latter contained a simpler and less morphologically accurate interface. Both phantoms were otherwise identical, with tissue-mimicking materials (TMMs) developed to mimic skin, subcutaneous fat, fibroglandular tissue, tumor and pectoral muscle. The phantoms’ construction used non-toxic materials, and they were inexpensive and relatively easy to manufacture. Both phantoms were scanned using conventional modalities (MRI, US, mammography and CT) and a recently introduced MWI radar detection procedure called in-coherent Multiple Signal Classification (I-MUSIC). Clinically realistic artifact-free images of the anthropomorphic breast phantoms were obtained using the conventional imaging techniques as well as the emerging technique of MWI.

## 1. Introduction

Physical anthropomorphic breast phantoms are essential tools for image quality assessment and optimization of imaging systems, recreating more realistic and controlled imaging scenarios [1]. They are often manufactured with highly realistic anatomical details to provide images with a similar appearance to in vivo body regions. Such phantoms can thus represent a ‘standard patient’ with known clinical truth, which can be subsequently employed for several useful purposes. For example, they can be used as tools in operator training for imaging or image-guided interventional procedures. They also represent a quality assessment and image optimization device for the initial implementation of an imaging protocol, and can be used for routine quality control of the same image modality to ensure that the scanning system is operating properly [2]. The use of anthropomorphic phantoms, with a high degree of anatomical, morphological and even pathological realism, is critical to ensure that these features are assessed in a realistically challenging scenario.

More importantly, realistic multi-modality phantoms are needed to assess against conventional modalities new promising diagnostic techniques such as microwave imaging (MWI) [3]. MWI methods can be grouped in Microwave Tomography [4,5,6] and Radar approaches [7,8,9,10,11]. While Microwave Tomography reconstructs the breast dielectric profile, Radar techniques only determine the location of strong microwave inhomogeneities within the breast. Regardless of the MWI method, the use of phantoms is critical to the clinical translation of microwave imaging devices from “bench to bedside”. Anthropomorphic breast phantoms which are capable of generating a realistic inter-tissue contrast for multiple imaging modalities will facilitate the assessment of MWI as breast screening solution in comparison with conventional diagnostics.

While sophisticated MRI-derived numerical breast phantoms have been developed (for example at the University of Wisconsin–Madison [12] and elsewhere), equally valid and universally-accepted experimental breast phantoms have yet to be developed and adopted for cross-modality investigation. Such phantoms are critical to the clinical translation of microwave imaging devices from “bench to bedside”. An appropriate and useful microwave breast phantom must meet important criteria such as anatomical and dielectric accurateness and durability. The creation of breast anthropomorphic phantoms capable of generating a realistic inter-tissue contrast for multiple imaging modalities will facilitate the assessment of MWI as breast screening solution in comparison with conventional diagnostics.

Improved anthropomorphic features were achieved by Mashal et al., who developed breast phantoms with four different breast densities: mostly fat, scattered fibroglandular, heterogeneously dense and extremely dense, for validation of MWI [13]. Skin, adipose tissue and a heterogeneous mix and fibroglandular tissue were made with oil-in-gelatin dispersions by varying the percentage of oil. For each phantom, the volume percentages of adipose tissue, fibroglandular tissue and heterogeneous mix changed. The phantoms exhibited accurate dielectric properties in the frequency bandwidth 1–6 GHz and good long-term stability. However, the phantoms were designed specifically for evaluating MWI and thus were not suitable for imaging with any other modalities. In another study, a realistic 3D-printed breast phantom for use in preclinical experimental microwave imaging studies was developed, which accurately reproduced adipose and fibroglandular tissue properties [14]. The adipose layer was simulated using 3D-printed plastic material, while the fibroglandular tissue was simulated by using liquid-filled voids in the plastic material. The 3D model for the phantom was MRI derived from a human subject leading to high anatomical accuracy. Furthermore, the phantom offered a good match to the dielectric properties of breast tissues in the frequency range between 500 MHz and 3.5 GHz, but here again no attempt was made to use this phantom with other imaging modalities. More recently, 3D-printed adjustable, realistic breast phantoms for microwave imaging [15] were disseminated among members of the European Cooperation in Science & Technology (COST) Action TD1301 “MiMed” [16] to establish a common standard to assess different image formation techniques.

Although several anthropomorphic multi-modality phantoms have been reported in the literature [17,18] with a handful of commercial multimodality phantoms developed in recent years (for example, by Yezitronix [19,20] and CIRS [21], these devices focus on the conventional modalities and have not been designed to mimic the dielectric properties which are suitable for MWI). Furthermore, these phantoms are designed to meet broad generic designs typically not based on patient-specific data and the tissue mimics are not customized for specific clinical applications. As a result, a number of different types of TMMs have been developed by research groups over the past two decades, typically manufactured to meet the specific physical properties of one or two imaging modalities, such as ultrasound or MR [22]. The main limitations associated with the different TMMs used to date has been their limited longevity in the case of gel matrix composition or the increased attenuation and low speed of sound in the case of rubber compositions [22]. Furthermore, there have been no studies to date which have investigated the development of tissue-mimicking materials which meet the requirements of the range of conventional clinical imaging but also the new imaging technique of MWI with the breast as the specific clinical application.

The aim of this study was to develop a novel anthropomorphic breast phantom capable of generating realistic patient images across all imaging modalities (ultrasound, MRI, CT, mammography and MWI) and enabling validation of the new MWI imaging technique. This involved the development, characterization and fabrication of anthropomorphic devices with five different breast tissue-mimicking materials (TMMs) (i.e., skin, subcutaneous fat, fibroglandular tissue, malignant tumor lesionand pectoral muscle), to provide a realistic match of the heterogeneity and morphological shape of the breast [18]. The developed TMMs were then incorporated into complex anthropomorphic phantoms, which are solid, ergonomic, easy to manufacture and avoid toxic materials such as formaldehyde. Finally, the prototyped breast phantoms were imaged by different modalities, including MWI.

The paper is organized as follows. In Section 2, the manufacturing and characterization protocols for the different TMMs are presented and the achieved tissue properties compared to the literature results. Section 3 and Section 4 detail the molds and the procedure used to create two breast phantoms, and Section 5 reports their images through standard modalities. In Section 6, the produced breast phantoms are imaged through. In this section, in particular, we supplement the MWI Radar approach that we presented in [10,11] with a novel clutter rejection procedure. Finally, discussion and conclusions complete the paper.

## 2. Tissue-Mimicking Materials

In this section, we report details concerning the TMMs manufacturing protocols and their characterization. Five different types of TMMs were developed as part of this study to mimic the behavior of skin, subcutaneous fat, fibroglandular tissue, pectoral muscle and tumor for the different energy interactions produced by the diagnostic imaging modalities of MRI, B-mode Ultrasound, Mammography, CT and Microwave Imaging. The properties of all the manufactured TMMs were fully optimized and characterized, including their magnetic resonance relaxation times, acoustic properties, X-rays linear attenuation coefficients and dielectric properties.

### 2.1. Reference Values for Breast Tissues

The dielectric characterization was carried out in terms of breast tissues relative permittivity and conductivity. The reference values for skin, fat and pectoral muscle were taken from Andreuccetti et al. [23], who reported relative permittivity and conductivity of human tissues across a large frequency range. The reference values for fibroglandular tissue and carcinoma (malignant tissue) were calculated from Debye model parameters provided by Lazebnik et al. [24,25], and Sugitani et al. [26]. Due to its heterogeneity, for the fibroglandular tissue a range of reference values was provided, rather than one single target value. For the sake of simplicity, Table 1 shows reference values at 2.5 GHz. However, a good match in dielectric properties was pursued in the frequency bandwidth between 500 MHz and 4 GHz. The acoustic characterization was carried out in terms of attenuation coefficient and speed of sound; reference values reported in Table 1 are taken from [27,28,29,30,31].

A realistic attenuation coefficient in breast tissue-mimicking materials provides realistic image and contrast detail. On the other hand, a realistic speed of sound provides accurate distance measurements. Note that no value was found in literature for attenuation in pectoral muscle. As for the magnetic resonance properties characterization, reference values for spin-lattice relaxation time (T_1_) and spin-spin relaxation time (T_2_) at 3 T are listed in Table 1 for subcutaneous fat, fibroglandular tissue [32] and pectoral muscle [33] For breast tumor tissue the only values identified in literature were for T_1_ and T_2_ at 1.5 T, although it is known that increasing the static magnetic field from 1.5 T to 3 T increases T_1_ and slightly decreases T_2_ [34]. For skin, no reference value was found in the literature. The X-ray attenuation characterization was carried out in terms of Hounsfield Unit (HU) measurements at 80 kVp, which was the minimum kV_p_ achievable with the available measurement set-up [35]. No reference HU values were found for skin and pectoral muscle.

### 2.2. Tissue-Mimicking Material Manufacturing Protocols

To develop the Tissue-Mimicking Materials (TMMs) for fibroglandular, tumor and pectoral muscle tissue, the initial stage was an agar-based International Electrotechnical Commission (IEC) TMM recipe [36]. This TMM was reported to have ideal acoustic and MR properties for mimicking human tissue [37]. The manufacturing process was similar for the skin, fibroglandular and pectoral muscle TMM recipes. The skin was made from a mixture of water- and Polyvinyl Alcohol Cryogel (PVAc)- based solution (“PVAc component”) and a fat component (“fat component”). This was based on a material that was previously developed by our group as a mechanically robust vessel-mimicking material (as reported by King et al. [37]) and a fatty liver tissue-mimicking material (as reported by Cournane et al. [38]). It was important that the skin mimicking material was mechanically robust, to provide long-term mechanical stability to the breast anthropomorphic phantom. The complete recipe is provided in Table 2. The fat component was used in the manufacture of the skin, fibroglandular and pectoral muscle TMMs. This was produced by combining 90 wt.% olive oil, 9 wt.% deionized water and 1 wt.% Synperonic A7 surfactant [39] and producing a uniform fat emulsion by thoroughly mixing the three components with a blender until the solution turned white and had a thick mayonnaise consistency.

The skin mimicking material was produced using an 80% PVAc component and a 20% fat component. The PVAc component was produced using 10 wt.% of 99 +% hydrolyzed poly(vinyl alcohol) powder (Sigma-Aldrich^®^ typical Mw 89,000–98,000) combined with 90 wt.% deionized water and 0.05 wt.% concentration benzalkonium chloride. Separately, the fat component was produced by mixing the surfactant and water together and heating to 50 °C, to ensure a homogenous solution, before adding olive oil and blending with the blender. Once blended, the fat component was added to the PVAc mixture and stirred until a homogenous mixture containing 20% by weight fat component was produced. The mixture was poured into the skin mold and allowed to cool to room temperature, and then left to rest for an additional two hours to allow for air bubbles removal. Solidification of the PVAc samples was induced by a series of 24-hour freeze/thaw cycles [38].

Materials and quantities used for the constituent fibroglandular, tumor and pectoral muscle agar-based TMMs are presented in Table 2. To produce the agar-based TMMs, the liquid components (water, glycerol and benzalkonium chloride) were mixed together in a stainless-steel container. The dry components (agar, SiC (17 µm), Al_2_O_3_ (3.0 µm) and Al_2_O_3_ (0.3 µm)) were weighed and then added to the liquid mixture. The stainless-steel container was then place in a heated water bath at 96 °C and an electronic overhead stirrer was used to continuously mix at 60 rpm and, thereby, maintain even particle dispersion within the mixture. A multimeter with an integrated temperature probe (Fluke 16, Eindhoven, Netherlands) was used to monitor the TMM mixture temperature to maintain it at 90 °C for one hour. The mixture was then removed from the water bath and allowed to cool at room temperature, while continuing to mix at 75 rpm using the overhead stirrer. When the temperature of the TMM had cooled to 46 °C, it was poured into either a specific mold for characterization purposes or the relevant breast mold as part of the phantom construction. For long-term storage, the TMMs were kept in an air-tight container with a mixture of water (87.67%)/glycerol (11.84%)/benzalkonium chloride (0.48%) at room temperature. This solution ensured the concentration of glycerol component in the TMM is maintained and prevented bacterial invasion [39].

The subcutaneous fat TMM was manufactured using a combination of a water mixture (deionized water (10.9%)/NaCl (0.11%) and a beeswax mixture (Pure Synperonic A7 Surfactant (9.8%)/safflower oil (31.7%)/beeswax (47.5%)). The specified quantity of beeswax was weighed and placed in a stainless-steel container and safflower oil was added. Deionized water was poured in a glass beaker together with the NaCl. The stainless-steel container containing the beeswax and safflower oil was placed into the water bath, the temperature of which was set to 94 °C. When the beeswax melted, the solution was homogenized with a whisk and the specified quantity of pure surfactant was added. The glass beaker containing the water and NaCl mixture was then put in the water bath and mixed with a whisk until NaCl dissolved. When the temperature of both the water and the beeswax mixture reached 80 °C, the water mixture was added to the beeswax mixture and stirred vigorously for five minutes. The stainless-steel container was then removed from the water bath and allowed to cool to 55 °C, while continuously stirring the mixture. Once the mixture reached 55 °C, it was poured into either a specific mold for characterization purposes or the relevant breast mold as part of the phantom construction.

### 2.3. Characterization Protocols

Samples of the five TMMs were made for characterization purposes. For the acoustic characterization, solid 1-cm thick samples were made while 2-cm thick samples were used for magnetic resonance relaxation times and X-rays linear attenuation coefficient measurements. For the dielectric characterization, liquid samples were obtained by mixing the ingredients without activating the gelling process by placing the TMM mixture in the water bath. The choice of using liquid samples was due to the simplicity of the chosen measurement setup. Furthermore, no evident difference in the dielectric properties was noticed when comparing liquid and solid samples.

The parameters of interest were hydrogen longitudinal and transverse relaxation times—T_1_ and T_2_, respectively. For the measurement of T_1_ and T_2_ relaxation times of the TMM samples, scans were performed using a two-point ratio method with a mixed sequence implemented on a 3T MRI system (Achieva, Philips Medical Systems, The Netherlands). Inversion recovery (IR) and spin echo (SE) sequences were used to determine T_1_ and T_2_ values, respectively. Potential phase errors were avoided due to the use of a phase correction built into the sequence. The scan parameters for the mixed sequence were as follows: resolution = 1.9 × 1.9 × 10 mm^3^, inversion time, TI = 800 ms, Time to Echo (TE) = (11, 22, 33, 44, 55, 66, 77, 88 ms), and repetition times were 6000 ms for the IR and 7000 ms for SE. A stack of all the samples was made and a statistically significant region of interest (ROI) was chosen to calculate a mean value of T_1_ and T_2_ for each layer.

The acoustic characterization was carried out using an in-house Scanning Acoustic Macroscope (SAMa) system, which was operated in pulse-echo configuration with a single transducer acting as both a transmitter and a receiver [39]. The employed broadband immersion transducer had a frequency range between 5.15 and 9.44 MHz and was centered at 7.5 MHz. All acoustic measurements were performed in deionized water at 20 ± 0.5 °C according to [40]. For the speed of sound and the attenuation coefficient characterization of each TMM sample, two datasets were acquired and processed: a reference signal and a sample signal. The reference signal was acquired from a scan of a reference glass reflector, while the sample signal was acquired with the sample between the glass reflector and the transducer [41]. The attenuation coefficient was determined by measuring pulse amplitudes when the sample was inserted in and absent from the path of the ultrasound beam.

To characterize the interaction between the TMMs and X-ray, the Hounsfield Unit (HU) was measured for all the samples. HU is a transformation of the linear attenuation coefficient μ and is provided by a CT scan of the samples together with a calibration phantom. The measurement of the Hounsfield number was performed using a 64-slice CT scanner (Sensation, Siemens Medical Solutions, Karlsruhe, Germany). HU were measured at 80 kVp, with a pixel size of 0.97 × 0.97 mm^2^. In order to maintain HU consistency, the scanner was calibrated using a phantom made of different reference materials with known attenuation coefficients, provided by the manufacturer. This was simultaneously used with the TMMs samples during the scan. This way, the calibration procedure is automatic, being the calibration phantom present in the axial Field of View (FOV). Once the volumetric scan was performed, a circular ROI for each TMM was selected and a mean value of the Hounsfield number was given for each ROI and TMM sample.

The setup for broadband measurement of dielectric properties of the TMM samples consisted of a vector network analyzer (VNA) (model ZVB8, Rhode & Schwartz, Germany; operating frequency band: 300 kHz–8 GHz), an open-ended coaxial probe and a coaxial cable used to connect the probe to the VNA. The probe was constructed from standard 6.3-mm diameter, 50-Ω, Teflon-filled, semi-rigid coaxial cable (RG401) whose inner conductor had a diameter of 1.63 mm, while the external shield had a mean diameter of 5.28 mm. The measurement was carried out following the measurement setup described in [42]. Four references were used for calibration: open circuit, short circuit and two reference liquids (i.e., deionized water and methanol). The complex reflection coefficient S_11_ was then measured for the four reference loads and for the TMM sample. After every measurement, the probe was cleaned by immersion into an isopropyl-alcohol solution. The measured reflection coefficients were processed by an in-house-developed MATLAB program based on the procedure proposed in [43].

### 2.4. Physical Properties Results

Measured physical properties were compared with the reference values previously reported in the literature. The aim was to achieve either a good match with the reference values or a clinically relevant contrast between different TMMs. In particular, the TMMs properties were optimized and refined to achieve a realistic contrast between adjacent layers such as skin-fat, fat-fibroglandular, fibroglandular-tumor and skin-pectoral muscle.

**Figure 1:**
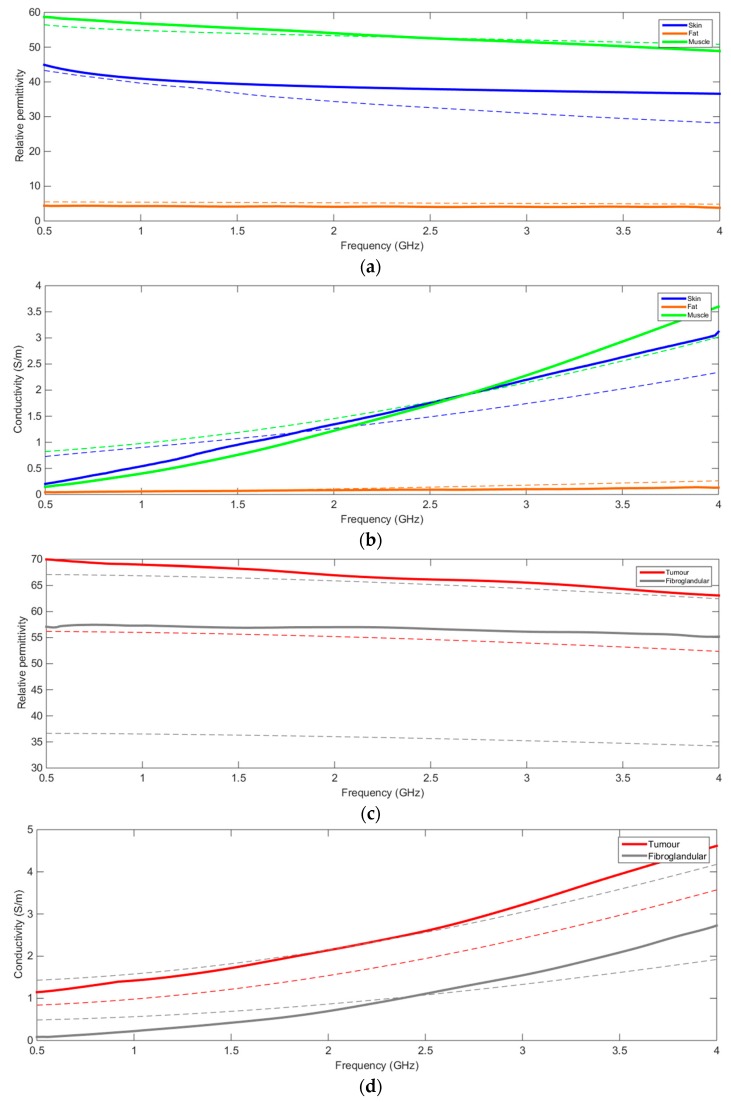
Comparison between measured (continuous line) dielectric values and reference values (broken line) for skin, fat, pectoral muscle, fibroglandular tissue and tumor TMMs. Relative permittivity (**a**) and conductivity (**b**) comparison for skin, fat, pectoral muscle and fibroglandular tissue TMMs. Two dashed lines are displayed for skin, fat and pectoral muscle tissue to represent the variability documented in the literature. Relative permittivity (**c**) and conductivity (**d**) comparison for tumor and fibroglandular TMM. Only one dashed line is displayed for the tumor tissue where less variability was reported [12,24,25].

Measured relative permittivity and conductivity of the TMM samples in the frequency bandwidth between 500 MHz and 4 GHz are presented in Figure 1. The measured values (continuous lines) were compared with the reference values found in the literature. Given the broad range of dielectric properties variability, for fibroglandular tissue the measured data of the relevant TMM are plotted against the minimum and maximum values reported in the literature. As can be seen, the reference and measured values of the electric conductivity and permittivity showed a good match for the skin, pectoral muscle and subcutaneous fat layers. This also resulted in an accurate conductivity and permittivity difference between these different tissue-mimicking layers, in particular, at the interface between skin and fat. However, the conductivity curves did not demonstrate stability over the entire frequency bandwidth (Figure 1). The conductivity curves for skin and muscle have a higher slope than the reference ones. Control of the conductivity over a large bandwidth is challenging and requires further investigation. Nevertheless, the overall difference in the conductivity values was realistic and stable. The TMMs were found to provide an acceptable match in the framework of multi-modality imaging feasibility which implies a trade-off between competing physical properties. An acceptable match was also observed for the reference and measured values of fibroglandular and tumor tissue. Specifically, the permittivity contrast was accurate, but the conductivity contrast was higher than the maximum reference value. However, it should be noted that the relatively low difference between the conductivity values of these two tissues was deemed to be sufficient as a starting point to provide the required contrast in the anthropomorphic phantom for appropriately challenging MWI techniques in a realistic manner, as the fibroglandular tissue was matched to the more challenging, dense fibroglandular tissue.

Table 3 lists the measured values of acoustic (i.e., speed of sound and attenuation coefficient), magnetic resonance relaxation (i.e., T_1_ and T_2_) parameters and HU.

Results in Table 3 show an excellent match (within ±10%) between reference and measured values for speed of sound (SOS) for all the layers, except for the fat layer the SOS of which was +15% higher than the reference value. However, the mismatch for the skin layer SOS is not significant and would not represent a distance artefact in the appearance of the skin in an ultrasound image as the skin layer in the anthropomorphic phantom was designed to be less than 2-mm thick. The measured attenuation coefficient of skin, fibroglandular and tumor tissue were lower than the literature reference values but were all within ±10% apart from the tumor which was significantly lower. However, the reference values do not take into account compression of breast tissues, which is not relevant to this work, as no compression was applied to the phantom during the imaging process. With compression the attenuation of the breast tissue is known to reduce, this is why breast tissue is compressed during an ultrasound examination if the tissue is too attenuating. Although, the attenuation coefficient achieved for these tissue mimics is lower than the reference values it is still relevant as it may approximate the attenuation coefficient value for in vivo compressed breast tissue [44]. The measured speed of sound value for the fat tissue mimic was 15% higher than the reference values for mammary fat, this would cause underestimation of distance within an ultrasound image. 

The reported magnetic relaxation results show an excellent match between reference and measured values (within ±10%) was achieved for all the tissue layers, except for the fibroglandular TMM. The T_1_ and T_2_ values for the fibroglandular tissue mimic were significantly lower than the reference values. Furthermore, the measurement for the fat TMM demonstrated some variability in the T_1_ and T_2_ values for the same sample. However, the range of values reported (300–521 ms for T_1_, 40–84 ms for T_2_) represent the range of values that were found for the relaxation properties of breast fat tissue mimic presented here. These results show that an addition of olive oil and surfactant to the IEC TMM (as it happened for the fibroglandular TMM) resulted in a lower T_1_ value, which decreased from 1504 ± 10 ms [32] to 624 ± 10 ms.

For X-ray characterization, the HU values were measured at 80 kVp. An excellent match (within ± 10%) was achieved the reference and measured values for fat. The fibroglandular and tumor tissues were found to have substantially higher HU values compared to the reference values. For the fat TMM, a high percentage of safflower oil and beeswax resulted in a low attenuation coefficient compared to water (HU = 0 for water). The same can be said for the fibroglandular layer, this was largely due to the addition of the olive oil and surfactant to the IEC TMM which caused a shift in the HU from 58.6 ± 7.3 [35] to 46.88 ± 23.90. Overall, the different breast multi-modality TMMs were found to provide a good approximation at matching the reference values with and the interaction of the different breast tissues types with the electromagnetic and sound energy to the conventional imaging techniques used for breast screening and imaging, mammography, CT X-ray, magnetic resonance and ultrasound. This approximation was very good given that seven parameters were involved in establishing that each of the five types of TMMs corresponded with the respective tissue types. Some deviation from the published values had to be accepted in order to make these materials useful for the four imaging modalities. Furthermore, the TMMs needed to be stable, robust and safe (for example, no ferromagnetic materials were used) so that they could be incorporated into anatomical phantoms which could produce artefact-free images (artefacts due to the TMMs) with adequate image contrast for all four imaging modalities.

## 3. Anatomical Breast Phantom Mold Generation

Two different types of breast phantoms were developed. The first (which will be referred to henceforth as ‘Phantom A’) had a simple and less morphologically accurate interface between mammary fat and fibroglandular tissue; the second (’Phantom B’) had a more relevant complex fat and fibroglandular interface, extracted from real patient MRI datasets. Notwithstanding the different shapes, the building process was the same for both phantoms. For each phantom, a set of 3D-printed molds was used; each set consisted of three molds: the external breast mold, the skin mold and the internal fibroglandular mold. Finally, a third silicone mold was used to shape the TMM tumor lesion.

For Phantom A, an ‘stp’ file was created by the surface scanning of the external shape of a hand-molded breast phantom. This file was imported in a numerical environment (CST Studio Suite Computer Simulation Technology, Germany) and used for printing the skin mold. The external breast mold was then obtained with the same file by Boolean subtraction. Finally, the internal fibroglandular mold was obtained from a scaling of the skin mold shape. Scaling the same shape gives a less anthropomorphically realistic phantom for the lack of a more typical intricacy of the fat/fibroglandular tissue boundary; however, it allows control of the volume ratio between fat and fibroglandular tissue, which may vary from breast to breast and may affect image quality. A sharp contrast between tumor and fat tissues of the relevant physical properties for the imaging modalities herein considered is observed in comparison to the tumor/fibroglandular tissue contrast. This corresponds to a more challenging scenario in the presence of very dense breasts in terms of fibroglandular tissue. This is particularly true for MWI due to the high average dielectric contrast of about 10:1 between tumor and fat tissue compared to a more challenging ratio of 1.5:1 between tumor and fibroglandular [13,25,26]. For this reason, the scaling factor was chosen in order to have adipose and fibroglandular tissue in a 50/50 ratio.

The molds for Phantom B were 3D printed from an MRI-derived stl file available in the University of Wisconsin Cross-Disciplinary Electromagnetics Laboratory (UWCEM) Numerical Breast Phantom Repository [14]. 

**Figure 2:**
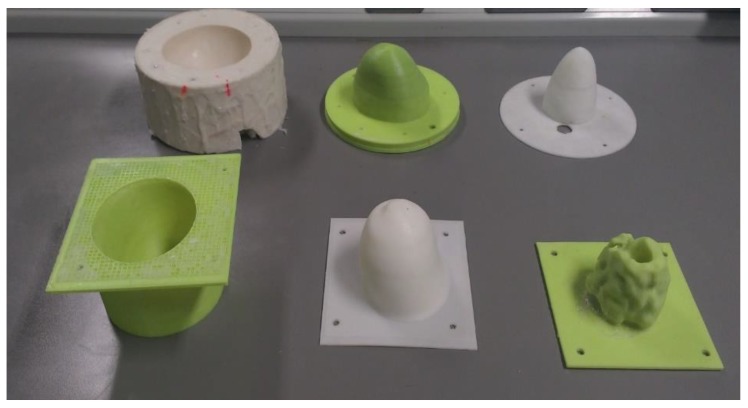
The molds used to make the anatomical multimodal breast phantoms. The top row shows molds for Phantom A. The bottom row shows the molds for Phantom B. From left to right: external breast molds (**left**), skin mold (**center**) and internal fibroglandular mold (**right**).

The main difference between the molds for Phantom A and B was in the shape of the internal fibroglandular mold, which makes the fibroglandular layer in Phantom B more anatomically realistic. The set of molds for Phantom A and B are presented in Figure 2.

To make the tumor targets, a two-part silicone mold was made by pouring silicone around metallic spheres of 1.5-cm diameter, to reproduce 1.5-cm TMM tumor lesions to place in the phantoms. The silicone molds presented regular spherical cavities where the tumor TMM was poured to produce tumor-TMM spheres which were finally embedded in the fibroglandular TMM of the phantoms. A single 1.5-cm diameter sphere was used in this study for each phantom.

## 4. Phantom Construction

**Figure 3:**
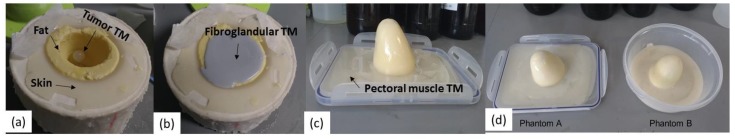
Phantom construction: (**a**–**d**).

Each breast phantom was constructed in five stages using the breast molds, an external breast mold, skin mold and internal fibroglandular mold. The external breast mold replicated the shape of a human breast in a prone position and the internal fibroglandular mold in Phantom B provided a structurally complex interface between the fat and fibroglandular regions. In the first stage of phantom construction, the tumor was set in a simple hemispherical mold. In the second stage, a thin skin layer (1–2 mm) was set using the external breast mold and the skin mold (Figure 3a). The skin layer was allowed to fully congeal for five hours before proceeding to the next stage. In the third stage, the subcutaneous fat layer was set using the skin mold as the external mold and the internal fibroglandular mold as the opposing internal mold. The subcutaneous fat layer was allowed to fully congeal for five hours before proceeding to the next stage. In the fourth stage, the tumor was suspended in the fibroglandular cavity and then the fibroglandular tissue mimic was poured into the cavity and allowed to fully congeal for five hours (Figure 3b). The final stage involved pouring the pectoral muscle tissue mimic into a fat tray and, once this tissue mimic was slightly congealed, the main breast phantom was sat into the slightly molten tissue mimic (Figure 3c). The final versions of Phantom A and B are presented in Figure 3d.

## 5. Conventional Imaging Results

The two breast phantoms were scanned with MRI, B-mode ultrasound, CT and mammography. As MWI is not a standard diagnostic technique, this imaging modality is discussed separately in the following section.

**Figure 4:**
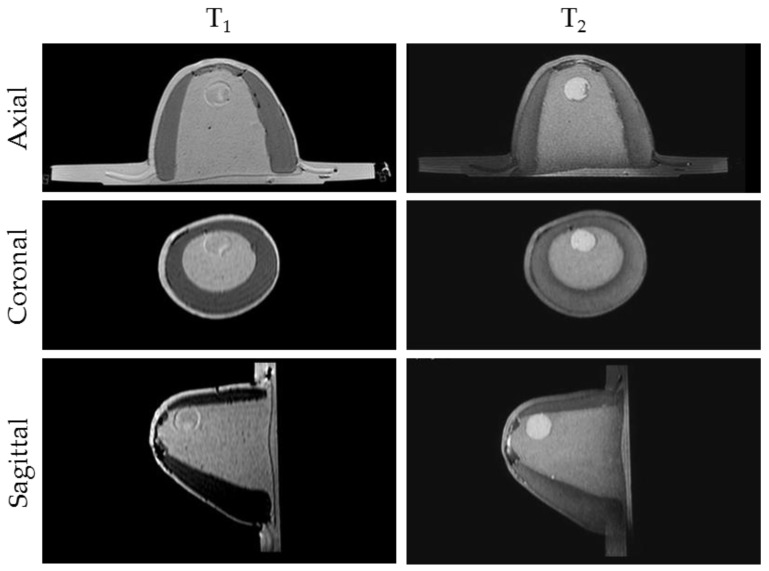
Representative MRI images of Phantom A. $T_{1}$-weighted images (**left**) exhibited a relatively poor contrast between the fibroglandular tissue and tumor, while the axial $T_{2}$-weighted images (**right**) exhibited improved contrast, as seen clinically.

**Figure 5:**
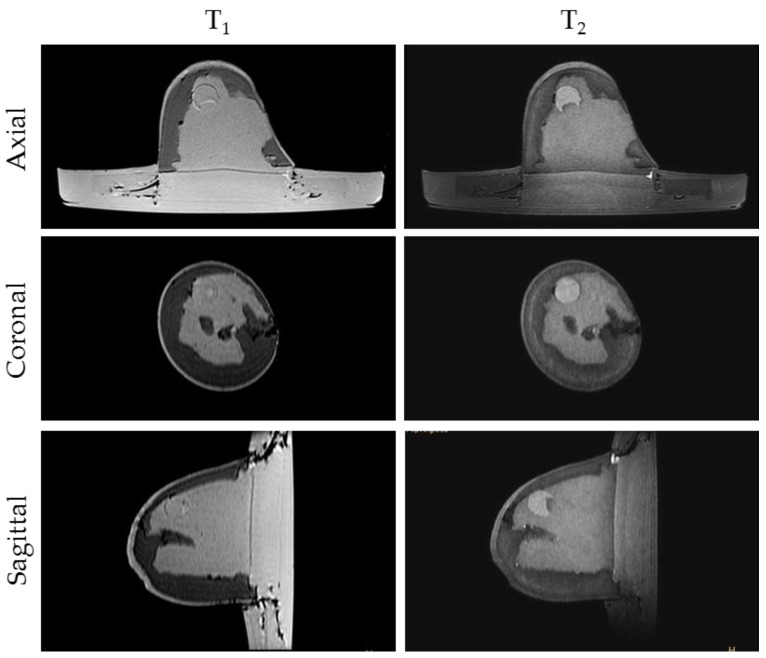
Representative MRI images of Phantom B. $T_{1}$-weighted images (left) exhibited a relatively poor contrast between the fibroglandular tissue and tumor, while the axial $T_{2}$-weighted images (right) exhibited improved contrast, as seem clinically.

**Figure 6:**
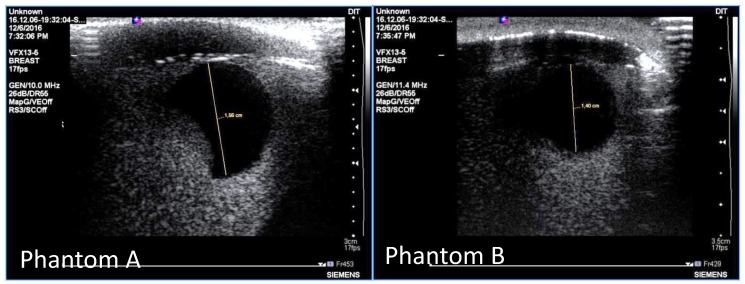
Representative ultrasound images of Phantom A (**left**) and Phantom B (**right**). The skin is the first layer, then the next hypoechoic layer is the subcutaneous fat mimic, the black hypoechoic region is the tumor mimic and the echogenic region surrounding the tumor is the fibroglandular tissue mimic.

**Figure 7:**
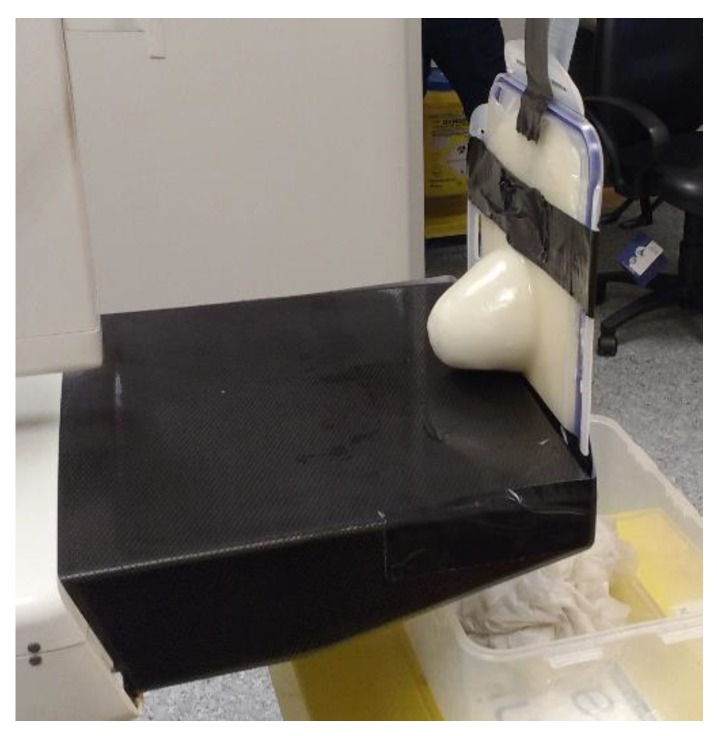
Photograph showing the placement of Phantom A for the mammography imaging. No compression was applied to the phantom.

**Figure 8:**
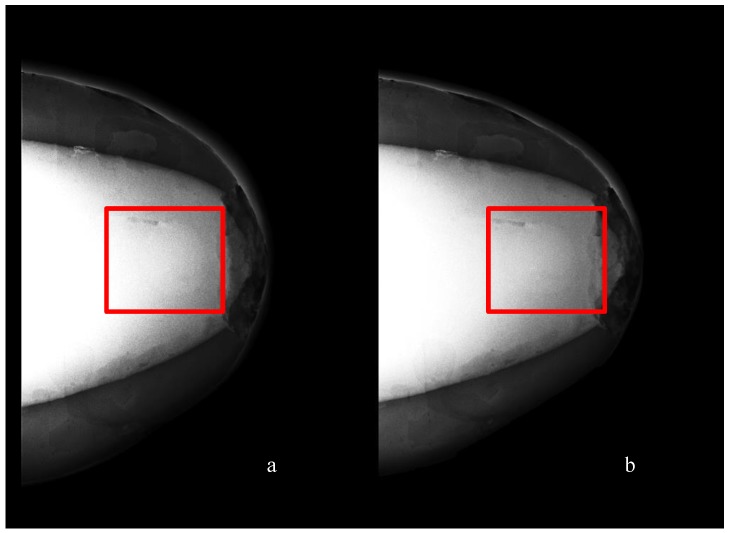
Representative mammography images of Phantom A obtained by using a W/Rh anode/filtration combination at (**a**) 28 kVp and (**b**) 34 kVp.

**Figure 9:**
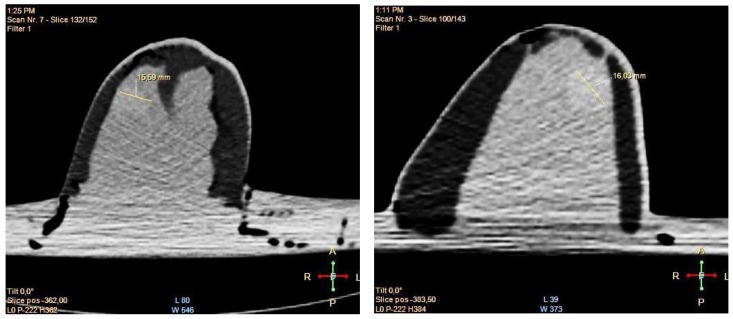
Representative CT images of Phantom A (**left**) and B (**right**).

- MRI scanning was performed using a 3T system (Achieva, Philips Medical Systems, Netherlands). A high-resolution 3D T_1_-weighted image was acquired with a turbo gradient echo sequence, with an echo time TE of 2 ms, a repetition time TR of 4 ms and a flip angle of 10°. The spatial resolution was 0.4 × 0.4 × 0.8 mm^3^. A high-resolution 3D T_2_-weighted image was acquired using a 3D turbo spin echo sequence, with a TE of 378 ms and TR 2.5 ms. Resolution was 0.34 × 0.34 × 0.8 mm^3^. The high spatial and contrast resolutions of the resultant images facilitated their use as a reference for the other imaging modalities for localizing and characterizing the shape and size of the tumor. The MR images obtained for Phantom A and B are presented in Figure 4 and Figure 5. A low contrast both in terms of T_1_ and T_2_ can be observed between the tumor and the fibroglandular layer. However, the shape of the tumor can be distinguished from the fibroglandular background because of a chemical shift artefact at the interface between the two TMMs. This low contrast is consistent with that obtained clinically between malignant and fibroglandular tissues, which usually requires the injection of a contrast agent to enhance the visibility of the tumor.
- B-mode ultrasound scanning was performed using an Antares scanner (Siemens, Germany) with a VFX13-5 linear array transducer at a nominal frequency of 10 MHz. The output power was set to maximum and three focal zones were positioned at 10, 15 and 20 mm, which represented the entire length of the lesion. The maximum depth and FOV was set to 35 mm, which is representative of clinically relevant depth for breast ultrasound imaging. Representative ultrasound images of Phantom A and B are presented in Figure 6. All the layers can be distinguished, except for the pectoral muscle layer, which does not intersect with the scanning plane as this was beyond the typical clinically relevant field of view. Moreover, the indents in the tumor shape in Phantom A were accurately depicted in the ultrasound image. A mismatch in terms of speed of sound between the reference value (1479 ± 32 m/s) and the achieved one (1710 ± 17 m/s) for the breast fat, resulted in an underestimation of the thickness of the fat layer in the ultrasound image. This feature also occurs in clinical imaging where Coopers ligaments are present at the interface between fat and fibroglandular tissue; more importantly an accurate clinical contrast was achieved between the fibroglandular tissue and the tumor, with the tumor mimic being presented as a hypoechoic region within the image [45].
- Digital mammography scanning of Phantom A was performed with a Mammomat Novation DR system (Siemens Medical Solutions, Germany). The phantom was vertically taped to the device to reproduce the position of the breast during a mammographic exam (Figure 7). However, no compression was applied to the phantom as the fat-mimicking material was not elastically compressible. As a result, a poor contrast was observed between tumor and fibroglandular tissue. Phantom A was imaged at Mo/Rh 28 kVp and 140 mAs (Figure 8a) and at Mo/Rh 34 kVp and 160 mAs (Figure 8b), with a pixel spacing of 0.07 × 0.07 mm^2^, focal spot 0.3 mm and FOV 286 × 233 mm. The relative lack of contrast between the tumor and fibroglandular tissue was as a result of the lack of breast compression used during the acquisition, due to the lack of compressibility of the phantom materials. However, the relative contrast displayed between the fibroglandular and the tumor TMMs with the slightly higher kVp was slightly better. The bright external surface in each image is the skin TMM, while the darker irregular structure next to the skin is the subcutaneous fat TMM. The saturated white region is the fibroglandular tissue and the tumor mimic is within the red square; the contrast definition between both of these tissue types was challenging, which was representative of the clinical situation where dense breasts are imaged. Finally, as mentioned above, there was slightly better contrast between the fibroglandular tissue mimic and the tumor mimic with the slightly higher kVp. The same low contrast appears in the CT scanning results, performed with a Symbia TruePoint SPECT-CT scanner (Siemens Medical Solutions, Germany). The CT images were acquired at 80 kVp and 36 mAs, with a pixel spacing of 0.97 × 0.97 mm^2^ and slice thickness of 2 mm. In both phantoms, the tumor was localized using the MRI datasets as reference (Figure 5). Representative CT images of the phantoms are presented in Figure 9. As for the mammography images, a similarly low contrast was observed between the tumor and fibroglandular tissue. Nevertheless, the CT images demonstrated superior resolution of the internal structures, for example, showing the subcutaneous fat TMM fingers penetrating inside the fibroglandular layer.

## 6. Microwave Imaging Results

**Figure 10:**
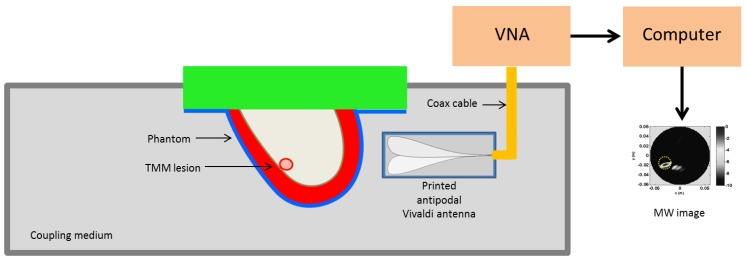
Schematic diagram showing the MWI scanning setup. The phantom is immersed in a coupling medium along with the antipodal printed Vivaldi antenna. The antenna is connected to a Vector Network Analyzer scanning the phantom at a fixed height in monostatic configuration. This allows collecting data for a single coronal slice.

MWI scans of phantoms were performed by using the measurement setup adopted in [46] and sketched for convenience in Figure 10. According to a multi-monostatic radar configuration, an antipodal Vivaldi antenna scanned the phantoms in the frequency range 0.5–3 GHz. In particular, the antenna scanned the phantoms at a given height by moving around them over 360° with a 5°-angular step (i.e., with 72 scanning positions). The MWI image formation procedure then returned a single 2D coronal slice reconstruction.

By adjusting the height, different coronal slices can be obtained. In general, data collected at different height can be simultaneously employed to get a 3D reconstruction. However, in this paper we exploited the sliced approach which is sufficient to assess the MWI detection capability. The phantom and the antenna were immersed within a coupling medium (with relative dielectric permittivity equal to 12). This was done for antenna miniaturization purposes and to reduce the dielectric discontinuity from the antenna side to the breast, which can hinder microwave energy penetration [11].

Images were obtained by first processing data by means of a clutter rejection algorithm aiming at mitigating the signal distortions coming due to the antenna’s internal reflection, the skin interface, and other non-tumor breast tissues. In particular, the algorithm was based on a hybrid artefact removal algorithm consisting of a two-step entropy computation and a subspace projection stage [46,47,48,49]. In more detail, the method allowed the setting of the time-gating window (before the signal is ruled out since assumed to be mainly clutter) and to select the subset of sensors’ positions where tumor contribution is stronger. Finally, reconstructions were obtained by processing the de-cluttered data though the MUSIC based method presented in [11].

**Figure 11:**
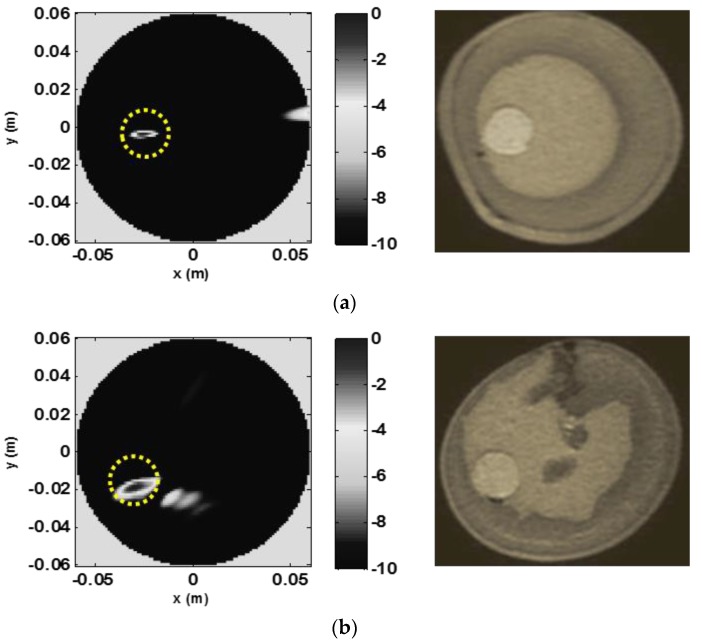
Representative MWI images (left) compared to the corresponding MR images (right). The yellow dotted circle represents the actual position of the tumor. (**a**) and (**b**) depict the images for phantoms A and B, respectively.

The obtained microwave 2D coronal slice reconstruction for phantom A (obtained from data collected at the height corresponding to the center of the tumor) is presented in Figure 11a (left) along with the corresponding MR image (right) for reference. In particular, according to the clutter-rejection procedure, the 2D image was obtained by setting the time gating at $t_{m_{min}}=3$ ns, and by exploiting only the sensors laying in the angular range between 135° and 275°. As expected from a multi-monostatic configuration which collects data only in reflection mode, the algorithm-selected signals come from the measurement circular sector which is closer to the tumor. The clutter-rejection procedure was then completed with the first projection discarded while performing the subspace projection.

**Figure 12:**
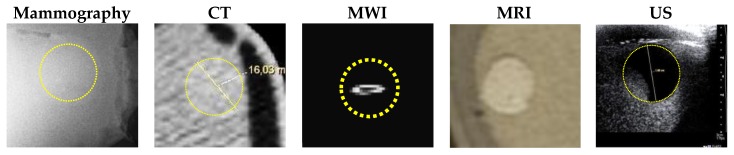
Image comparison of the TMM tumor in phantom A across the investigated modalities.

As can be seen in Figure 11 and Figure 12, MWI succeeded in detecting and locating the tumor. However, by comparing to the MR reconstruction, it can be observed that the MWI image does not represent the complex breast morphology or the shape of the tumor. This is due to the radar approach followed in the adopted MWI procedure which is only designed for detection purposes. Moreover, the clutter rejection method erased the contribution due to the skin layer and partially to the internal morphological features of the breast. This explains why the skin contour and internal details of the breast phantom were not reproduced with the circular mask in the image representing the contour of the scanned spatial region. The MWI procedure succeeds in detecting the tumor in both phantoms, including phantom B, which presents a more complex internal structure (Figure 11b). For phantom B, the algorithm returned the same time gating as for phantom A and almost the same observation angular range. However, in order to further reduce clutter coming from the internal mostly fibroglandular part of the phantom, the first two projections were discarded while performing the subspace projection. This could be expected as the investigated phantoms have different structures, with phantom B being more complex from a morphological point of view than phantom A and exhibiting less circular symmetry in the coronal cut considered. 

## 7. Conclusions

Clinical acceptance of an emerging imaging technology requires a rigorous validation process to ensure safety, efficacy and accuracy. Such process requires extensive clinical trials that can be expensive, lengthy and need to deal with the associated ethical challenges [50]. Preliminary, controlled, pre-clinical investigation can largely mitigate risks and costs associated to clinical studies. For this reason, together with suitable models, controlled synthetic phantoms are very valuable especially when they are suitable for more than one imaging modality.

In this paper, novel TMMs were developed and integrated into two phantoms which reproduced the main physical properties and parameters of the different types of breast tissue, such as dielectric, acoustic, relaxation times and X-ray attenuation coefficients, associated with different imaging techniques. A good match between the reference and measured respective physical properties, typically ±10%, was achieved for the majority of the TMMs, namely, skin, subcutaneous fat pectoral muscle and tumor layers. It is very challenging to create tissue mimics that can match all important parameters for different imaging modalities. However, it should be noted that, given the variations among and within real tissues, tissue mimics and phantoms have an important role as consistent targets for multimodality cross-calibration and training that human subjects and cadavers cannot serve. As a result of this, good agreement between reference and measured properties was demonstrated in the realistic images achieved across the different conventional modalities typically utilized for breast imaging as well as offering a promising reference TMM and phantom structure for the evaluation of MWI. In particular, the anthropomorphic phantoms produced highly realistic MR, mammography and CT images with excellent spatial resolution and high tissue contrast in the ultrasound images. These image features are immediately familiar to radiologists, thereby providing an introduction to MWI through the use of more familiar conventional images with known structures of which corresponding new microwave images can be obtained and reviewed. These new anthropomorphic phantoms can facilitate a greater confidence in the interpretation of the healthy tissue and benign and malignant pathology seen in the MW images.

## Figures and Tables

**Table 1:** Reference values for breast tissues properties.

	Dielectric Properties		Acoustic Properties		Magnetic Resonance Relaxometry Properties		X-Ray Attenuation Properties
	Relative Permittivity at 2.5 GHz	Conductivity (S/m) at 2.5 GHz	Speed of Sound ($m\cdot s^{-1}$)	Attenuation Coefficient at 7 MHz ($dB\cdot cm^{-1}\cdot mHz^{-1}$)	Spin-Lattice Relaxation Time $T_{1}\;\left(ms\right)$ @ 3T	Spin-Spin Relaxation Time $T_{2\;}\left(ms\right)$ @ 3T	HU at 80 kV_p_
Skin	38.007 [23]	1.464 [25]	1537 [29]	1.84 ± 0.44 [31]	*(No reference)*	*(No reference)*	*(No reference)*
Subcutaneous Fat	5.1467 [25]	0.137 [25]	1479 ± 32 [30]	0.6 ± 0.1 [31]	367 ± 8 [32]	53 ± 2 [32,33]	−102 ÷ −80 [35]
Fibroglandular tissue	35.7–65.3 [26,27]	1.52–2.37 [26,27]	1553 ± 35 [30]	2.0 ± 0.7 [31]	1445 ± 93 [32]	54 ± 9 [33]	12 ÷ 56 [35]
Carcinoma	54.69 [28]	1.89 [28]	1550 ± 35 [30]	1.0 ± 0.2 [31]	876 ± 28 at 1.5T [33]	75 ± 4 at 1.5T [33]	23 ÷ 78 [35]
Pectoral Muscle	52.729 [25]	1.7388 [25]	1545 ± 5 [30]	*(No reference)*	607 ± 85 [33]	36 ± 7 [33]	*(No reference)*

**Table 2:** Recipe for the skin, fibroglandular, tumor and pectoral muscle TMMs. All quantities in grams.

	Skin	Fibroglandular	Tumor	Pectoral Muscle
Polyvinyl Alcohol Cryogel	80	-	-	-
Agar	-	24	30	27
SiC	-	4.24	-	-
Al_2_O_3_ (3 μm)	-	7.6	-	-
Al_2_O_3_ (0.3 μm)	-	7.04	-	-
Sugar	480	-	200	360
NaCl	-	-	10	-
Glycerol	-	89.7	-	-
Deionized water	720	663.8	8.73	873
Benzalkonium Chloride	4	3.7	4.14	4.14
10% Synperonic A7 Surfactant	40	40	-	20
Olive Oil	160	160	-	80

**Table 3:** Measured values for acoustic, magnetic resonance relaxation and HU properties.

	Speed of Sound ($m\cdot s^{-1}$) @ 7.5 MHz	Attenuation Coefficient ($dB\cdot cm^{-1}\cdot mHz^{-1}$) @ 7.5 MHz	$T_{1}$ (ms) at 3T	$T_{2}$ (ms) at 3T	HU at 80 kV_p_
Skin	1657 ± 1 ***(+8%)***	1.77 ± 0.01 ***(+4%)***	292 ± 6 ***(No reference)***	33 ± 0.5 ***(No reference)***	100.75 ± 28.9 ***(No reference)***
Fat	1710 ± 17 ***(+15%)***	4.16 ± 0.05 ***(+593%)***	300–521 ***(Within range)***	40–84 ***(Within range)***	−108.78 ± 17.2 ***(+7%)***
Fibroglandular tissue	1532 ± 4 ***(−1%)***	0.73 ± 0.01 ***(−22%)***	624 ± 10 ***(−57%)***	35.5 ± 0.6 ***(−36%)***	46.88 ± 23.9 ***(+280%)***
Tumor	1573 ± 1 **(+1.5%)**	0.09 ± 0.01 ***(−91%)***	915 ± 14 ***(+4%)***	36 ± 0.6 ***(−50%)***	65.94 ± 31.5 ***(+183%)***
Pectoral Muscle	1577 ± 2.5 **(+2%)**	0.29 ± 0.01 ***(No reference)***	786 ± 20 ***(Within range)***	39 ± 1 (+8%)	52.25 ± 25.6 ***(No reference)***
